# Management and Surveillance of Immune Checkpoint Inhibitor Myocarditis: Managing the Aftermath: *JACC CardioOncology* State-of-the-Art Review

**DOI:** 10.1016/j.jaccao.2026.09.003

**Published:** 2026-10-20

**Authors:** Tyler T. Morad, Mohammad Keykhaei, Devon A. Terraciano, Pratyaksh K. Srivastava, Eric H. Yang, Ashley F. Stein-Merlob

**Affiliations:** a Department of Medicine, University of California at Los Angeles, Los Angeles, California, USA; b Division of Cardiology, Department of Medicine, University of California at Los Angeles, Los Angeles, California, USA; c UCLA-Cardio-Oncology Program, Division of Cardiology, Department of Medicine, University of California at Los Angeles, Los Angeles, California, USA; d San Diego Cardiac Center Medical Group, San Diego, California, USA

**Keywords:** arrhythmia, biomarkers, heart failure, immunotherapy, myocarditis

## Abstract

Immune checkpoint inhibitor (ICI)–associated myocarditis remains one of the most serious immune-related adverse events and can present major challenges after hospital discharge. Although guidelines and expert consensus statements have addressed diagnostic and management strategies in the acute phase, there is a paucity of evidence for post-discharge management and how to address recrudescence of symptoms and/or biomarkers suggesting rebound myocarditis. This review synthesizes evidence on the pathophysiology of ICI myocarditis, including the T cell–macrophage inflammatory loop that sustains smoldering disease, and provides a framework for outpatient management. We address steroid tapering strategies, the taper-flare phenomenon, and evidence for second-line agents and emerging therapies. Practical guidance is provided for biomarker surveillance, cardiac imaging protocols, antimicrobial prophylaxis, and the balance between immunosuppression and infection risk. This review aims to highlight and address significant gaps in cardio-oncology practice by offering clinicians an evidence-informed roadmap for managing this complex patient population.

## Highlights

• Outpatient management of steroid-refractory ICI myocarditis lacks evidence-based guidelines and standardized protocols.
• A biomarker-guided surveillance framework with structured steroid tapering and escalation triggers is proposed.
• Second-line agents, including abatacept and ruxolitinib, show promise but lack randomized comparative data.
• Prospective registries and randomized trials specifically addressing outpatient management strategies are urgently needed.

Immune checkpoint inhibitors (ICIs) have fundamentally transformed cancer treatment. Since the Food and Drug Administration (FDA) approved the first ICI agent, ipilimumab, in 2011, the therapeutic landscape has expanded rapidly, with more than 15 ICIs targeting proteins CTLA-4, PD-1, PD-L1, and LAG-3 now approved across more than 40 oncologic indications at the time of this writing.^1^ However, ICIs have introduced a broad spectrum of immune-related adverse events (irAEs) affecting nearly every organ system.^2^^,^^3^

Among these toxicities, ICI-associated myocarditis is uncommon but can have fatal complications. The reported incidence ranges from 0.04% to approximately 1%, depending on the treatment regimen and surveillance strategy, yet mortality remains disproportionately high, with published estimates ranging from 20% to 50%.^2^^,^^3^ This combination of low incidence and high fatality has made myocarditis one of the most important cardiovascular complications of ICI therapy to recognize and initiate timely treatment.

The acute management of ICI myocarditis has been increasingly discussed and studied in the cardio-oncology literature, with multiple professional societies recommending prompt ICI discontinuation, hospital admission with telemetry, and early initiation of high-dose intravenous corticosteroids, whereas steroid-refractory cases may require second-line immunosuppressive agents.^1^^,^^4^, ^5^, ^6^, ^7^, ^8^ However, risk stratification models, management recommendations, and current ongoing randomized clinical trials remain focused largely on inpatient acute management, with evidence to guide post-discharge care limited.

This gap is particularly important because many patients who survive the acute phase enter a prolonged outpatient course characterized by steroid tapering, serial biomarker surveillance, possible escalation to additional immunosuppressive therapies, and the ongoing need to balance disease control against infection risk and malignancy progression. These challenges are especially pronounced in patients with steroid-refractory or smoldering myocarditis, in whom troponin recrudescence may occur despite relative clinical stability. Additional challenges adding to this complexity include co-management of other ongoing toxicities, especially in the setting of triple M overlap syndrome involving muscular and neuromuscular toxicities. In this review, after a brief overview of the pathophysiology, diagnosis, and acute inpatient management of ICI myocarditis, we focus on the data and practical recommendations for outpatient care, including transition from inpatient management, early and late surveillance, steroid tapering, second-line immunosuppressive therapies, and infection prevention.

## Pathophysiology and Mechanisms of ICI Myocarditis

Under physiological conditions, immune checkpoints maintain cardiac immune tolerance. PD-L1, constitutively expressed on cardiomyocytes, engages PD-1 on T cells to suppress myocardial inflammation, whereas CTLA-4 restrains T-cell priming via CD80/CD86 interactions on antigen-presenting cells (Figure 1, left panel).^9^^,^^10^ ICI therapy disrupts these inhibitory pathways: anti-PD-1 antibodies permit T-cell activation and tumor killing, but activated CD8^+^ T cells may also mount an off-target immune response against cardiomyocytes sharing antigenic epitopes. α-Myosin heavy chain, which escapes central tolerance owing to its absence from the thymus, has been identified as a candidate autoantigen in a murine and human study, though it may not be a universal T-cell target across all cases of ICI myocarditis.^11^ Combined checkpoint blockade (anti-CTLA-4 plus anti-PD-1 or anti-LAG-3 plus anti-PD-1) amplifies this risk by removing redundant tolerance mechanisms.^9^^,^^10^

**Figure 1:**
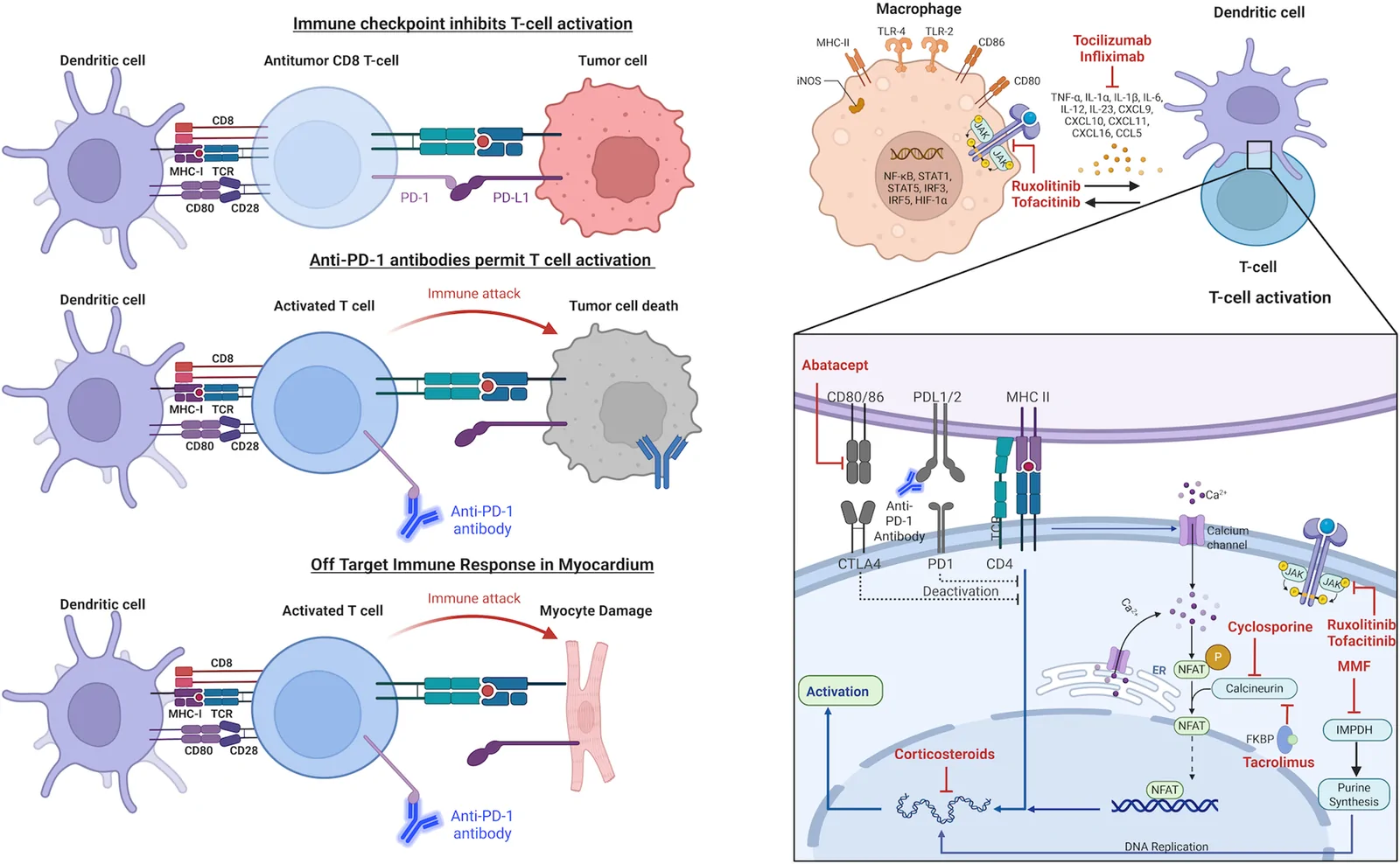
ICI Myocarditis Pathogenesis and Immunomodulatory Targets (Left) Checkpoint-mediated immune tolerance, immune checkpoint inhibitor (ICI)–induced antitumor immunity, and off-target myocardial injury. (Right) Immunomodulatory targets in both macrophages and T cells, including abatacept, corticosteroids, calcineurin inhibitors, ruxolitinib, tofacitinib, mycophenolate mofetil (MMF), tocilizumab, and infliximab. Ruxolitinib and tofacitinib are depicted as modulating both macrophage and T-cell signaling. Arrows indicate activation/signaling, and blunt-ended lines indicate inhibition. ER = endoplasmic reticulum; IMPDH = inosine monophosphate dehydrogenase; iNOS = inducible nitric oxide synthase; JAK = Janus kinase; MHC = major histocompatibility complex; NFAT = nuclear factor of activated T cells; NF-κB = nuclear factor kappa B; TCR = T-cell receptor; TLR = toll-like receptor.

Created with biorender.com.

The resulting myocardial injury is sustained by a self-amplifying T cell–macrophage inflammatory loop. Single-cell RNA and T-cell receptor sequencing have identified clonally expanded, cytotoxic CD8^+^ effector-memory populations in patients with ICI myocarditis.^12^ Interferon gamma from these effector T cells drives expansion of CCR2^+^ monocyte-derived macrophages expressing CXCL9 and CXCL10, which recruit additional CXCR3^+^ T cells to the myocardium.^13^^,^^14^ Depletion of either CD8^+^ T cells or macrophages mitigates disease, confirming that this bidirectional interaction is essential for pathogenesis. This self-perpetuating cycle underlies steroid-refractory and smoldering myocarditis: partial immunosuppression may reduce systemic inflammation without disrupting the tissue-resident loop, explaining troponin rebound during steroid tapering even in clinically stable patients.

These pathogenic mechanisms inform the therapeutic targets illustrated in Figure 1 (right panel). Abatacept (CTLA-4-Ig), blocks CD80/CD86-CD28 co-stimulation required for T-cell activation.^15^ Corticosteroids broadly inhibit proinflammatory gene transcription. Calcineurin inhibitors (tacrolimus, cyclosporine) prevent NFAT-mediated T-cell activation.^16^ Mycophenolate mofetil (MMF) inhibits inosine 5′-monophosphate dehydrogenase, depleting guanosine nucleotides essential for lymphocyte proliferation.^17^ Janus kinase (JAK) inhibitors (ruxolitinib, tofacitinib) target the JAK/signal transducers and activators of transcription (STAT) signaling cascade that amplifies inflammatory cytokine production in both macrophages and T cells.^18^ Tocilizumab blocks interleukin (IL)-6 signaling by binding soluble and membrane-bound IL-6 receptors, inhibiting the JAK/STAT3 inflammatory cascade that is significantly upregulated in ICI myocarditis.^19^ Genetic susceptibility, including human leukocyte antigen risk alleles (notably the 8.1 ancestral haplotype) and preexisting subclinical cardiac autoimmunity, may determine why only a minority of patients treated with ICIs develop myocarditis.^20^

### Summary

• A self-amplifying T cell–macrophage inflammatory loop sustains steroid-refractory and smoldering myocarditis.
• Therapeutic targets include co-stimulation blockade, JAK/STAT inhibition, calcineurin inhibition, and IL-6 receptor blockade.

## Clinical Presentation: The Spectrum of Disease

### Timing and onset

ICI myocarditis typically manifests early in the treatment course, with a median onset of 30 to 34 days after ICI initiation, often after 1 or 2 doses.^3^^,^^7^ The risk is significantly higher with combination checkpoint blockade: dual anti-CTLA-4 and anti-PD-1 therapy confers an approximately 4.7-fold increased risk compared with monotherapy, and anti-LAG-3 plus anti-PD-1 combinations may carry similarly elevated risk.^3^^,^^7^ Late-onset cases beyond 3 months have been reported, necessitating ongoing vigilance.

### Clinical spectrum: from subclinical to fulminant

The clinical presentation spans a broad spectrum. Symptoms are often nonspecific, including chest pain, dyspnea, fatigue, palpitations, syncope, and weakness, and some patients are identified solely through routine biomarker surveillance.^1^^,^^4^ Fulminant myocarditis manifests with hemodynamically significant arrhythmias, high-grade atrioventricular block, or cardiogenic shock. Notably, conduction abnormalities rather than pump failure can be the primary cause of death in fatal cases, and left ventricular ejection fraction (LVEF) is frequently preserved.^1^^,^^4^ Multiple grading systems have been developed to classify disease severity, including the American Society of Clinical Oncology 4-tier system, the International Cardio-Oncology Society (IC-OS) clinically actionable classification (smoldering, nonsevere, and severe/fulminant), and the Common Terminology Criteria for Adverse Effects, which are summarized in Table 1.^4^^,^^6^ After initial improvement, in a subset of these patients, their condition may evolve into a smoldering phenotype, defined by the IC-OS as troponin elevation meeting diagnostic criteria without clinical signs or symptoms.^6^ These patients may have preserved or recovered LVEF yet exhibit persistently elevated troponin, creating a clinical dilemma regarding immunosuppression intensity and readmission thresholds. This smoldering phenotype is thought to be sustained by the macrophage–T cell inflammatory loop (see “Pathophysiology and Mechanisms of ICI Myocarditis”) and constitutes a significant outpatient challenge.

**Table 1:** Severity Classification of ICI-Associated Myocarditis

Grade	Clinical Features
ASCO Grading System^4^	
1	Asymptomatic patients with abnormal ECG or troponin
2	Symptomatic patients with abnormal ECG and troponin
3	Symptomatic patient with findings on echocardiogram or CMR that are diagnostic or suggestive of ICI myocarditis
4	Life-threatening disease with cardiac abnormalities appreciated in grades 1-3
IC-OS Classification^6^	
Smoldering	Troponin elevation meeting diagnostic criteria without clinical signs or symptoms
Nonsevere	Symptomatic myocarditis without hemodynamic compromise
Severe (fulminant)	Hemodynamic instability, life-threatening arrhythmias, or cardiogenic shock
CTCAE^59^	
1	Mild; asymptomatic or mild symptoms; clinical or diagnostic observations only; intervention not indicated
2	Moderate; minimal, local, or noninvasive intervention indicated; limiting age-appropriate instrumental activities of daily living
3	Severe or medically significant but not immediately life-threatening disease; hospitalization or prolongation of hospitalization indicated; disabling; limiting self-care activities of daily living
4	Life-threatening consequences; urgent intervention indicated
5	Death related to adverse event

The ASCO system grades ICI-associated myocarditis from asymptomatic biomarker or ECG abnormalities to life-threatening disease. The IC-OS system classifies myocarditis as smoldering, nonsevere, or severe according to symptoms and hemodynamic status. CTCAE provides a general adverse-event severity framework from mild disease to death.

ASCO = American Society of Clinical Oncology; CMR = cardiac magnetic resonance; CTCAE = Common Terminology Criteria for Adverse Events; ECG = electrocardiogram; ICI = immune checkpoint inhibitor; IC-OS = International Cardio-Oncology Society.

### Overlap syndromes

The triple M overlap syndrome (concurrent myocarditis, myositis, and myasthenia gravis–like illness) is increasingly recognized, with approximately one-third of patients with ICI-associated myocarditis having concomitant myositis.^21^ For patients with triple M overlap syndrome, in-hospital mortality is 38% to 60%, primarily from respiratory muscle failure.^5^^,^^22^ The National Comprehensive Cancer Network (NCCN) recommends evaluating for concomitant myositis and myasthenia gravis–like symptoms in all suspected cases, with biomarkers including creatine phosphokinase (CPK), aldolase, and acetylcholine receptor antibodies.^7^ Notably, acetylcholine receptor antibodies are positive in only a minority of cases (26% in 1 cohort), and there is ongoing debate regarding whether these presentations represent true myasthenia gravis or a myasthenia gravis–like phenotype driven by myositis with a predilection for oculo-bulbo-respiratory muscles, which may account for the frequently negative serologies.^23^ Simultaneous elevation of CPK, aspartate aminotransferase, alanine aminotransferase, lactate dehydrogenase, and troponin portends the highest mortality.^5^ This biomarker profile informs outpatient risk stratification, and patients with concurrent myositis or myasthenia gravis–like syndrome require monitoring of respiratory function (negative inspiratory force, vital capacity) in addition to cardiac biomarkers.^7^^,^^22^

### Summary

• ICI myocarditis most commonly manifests within the first month of ICI initiation, with higher risk in combination regimens.
• It is critical to identify associated noncardiac irAEs. Concomitant myositis and myocarditis occur in up to one-third of patients and the triple M overlap syndrome (myocarditis, myositis, myasthenia gravis–like illness) is associated with mortality rates of up to 60%.
• Smoldering myocarditis with biomarker-symptom dissociation represents a major outpatient management challenge.

## Diagnosis

Diagnostic criteria for ICI myocarditis have been extensively addressed in contemporary society guidelines and consensus statements, and a detailed diagnostic algorithm is beyond the scope of this review. Briefly, diagnosis relies on integration of ICI exposure timing, symptoms, findings on electrocardiogram (ECG), cardiac biomarkers, and multimodality imaging, with endomyocardial biopsy reserved for cases in which noninvasive evaluation is inconclusive and tissue confirmation would meaningfully affect oncologic or immunosuppressive management. Notably, biopsy sensitivity may be reduced after immunosuppression is initiated, and fulfillment of IC-OS criteria with exclusion of acute coronary syndrome may be sufficient to establish the diagnosis in many patients.^6^ Troponin elevation remains central to diagnosis, though interpretation should account for overlap myositis, as cardiac troponin T (cTnT) may reflect skeletal muscle involvement, whereas cardiac troponin I (cTnI) is more cardiac specific. Lehmann et al.^24^ demonstrated re-expression of *TNNT2* (encoding cTnT) in regenerating skeletal muscle, providing a mechanistic basis for the disproportionate cTnT elevation seen with concurrent myositis. In this cohort, cTnT was elevated in more cases (94%-100% vs 82%-83% for cTnI), normalized more slowly (median 133 days vs 17 days), and may carry greater prognostic value for major adverse cardiovascular events, with implications for both screening and longitudinal surveillance.

Cardiac magnetic resonance (CMR) (with both T1 and T2 sequences) is the preferred noninvasive modality when feasible, whereas echocardiography and fluorodeoxyglucose positron emission tomography (FDG-PET) may provide complementary information in selected patients; however, these imaging modalities have limited sensitivity, and endomyocardial biopsy may be warranted for equivocal findings.^6^^,^^25^ Because symptoms are frequently nonspecific, and some cases are identified solely through biomarker screening, multiple professional societies have proposed on-treatment surveillance strategies to facilitate early detection, including baseline cardiac assessment and serial biomarker monitoring during the highest-risk period of ICI therapy. These recommendations vary regarding specific biomarkers, testing frequency, and risk stratification approach (Table 2).^4^, ^5^, ^6^, ^7^ In a prospective study by Cheng et al,^26^ investigators used cTnI surveillance and symptom-based triaging to facilitate early evaluation and treatment of cardiotoxicities, including myocarditis. However, because ICI myocarditis is rare and troponin elevation is not specific, routine troponin surveillance carries a low positive predictive value, with most elevations reflecting alternative causes; the optimal on-treatment strategy therefore still remains unclear, and no single approach has been prospectively validated to improve clinical outcomes.

**Table 2:** Society Recommendations for ICI Myocarditis Management

Domain	ASCO (2021)^4^	ESC (2022)^1^	NCCN (2025)^7^	ACC (2025)^5^	IC-OS (2026)^6^
IV steroid (Methylprednisolone) dosing	1000 mg/d	500-1000 mg for ≥3d	1 g/d for 3-5 d	500-1,000 mg/d for ≥3 d	500-1,000 mg/d
Oral steroid (Prednisolone) transition	1-2 mg/kg/d	1-2 mg/kg/d	1 mg/kg/d	Not explicitly defined	1-2 mg/kg/d
Taper duration	≥4-6 wk	4-6 wk	6-12 wk guided by biomarkers	Guided by troponin and clinical status	∼10 mg/wk; slower below 10 mg/d; guided by troponin
Severity classification	Grade 1-4	Fulminant (Non Fulminant/steroid recovery)	Grade 1-4	Not explicitly defined	Smoldering/nonsevere/severe (fulminant)
Baseline monitoring	ECG, troponin before each cycle	ECG, troponin, natriuretic peptides in all patients (class I, LOE B); echocardiography in high-risk patients	Consider hs-troponin and NT-proBNP at baseline and serially; hs-troponin every cycle × 3 cycles (high-risk), then every 3 mo	hs-troponin; CPK, AST, ALT biweekly × 12 wk (under investigation)	Troponin at baseline and before each cycle
Post-discharge monitoring	Not explicitly defined	Not explicitly defined	Follow-up with oncologist and subspecialists; topical patch monitoring outpatient	Not explicitly defined	Troponin-guided taper

Recommendations from 5 professional societies are compared across corticosteroid dosing, tapering, severity classification, baseline surveillance, and post-discharge monitoring. Substantial variation remains, particularly regarding steroid taper duration and outpatient follow-up.

ACC = American College of Cardiology; ALT = alanine aminotransferase; ASCO = American Society of Clinical Oncology; AST = aspartate aminotransferase; CPK = creatine phosphokinase; ECG = electrocardiogram; ESC = European Society of Cardiology; hs-troponin = high-sensitivity troponin; ICI = immune checkpoint inhibitor; IC-OS = International Cardio-Oncology Society; LOE = level of evidence; NCCN = National Comprehensive Cancer Network; NT-proBNP = N-terminal pro–B-type natriuretic peptide.

### Summary

• Diagnosis integrates ICI exposure timing, biomarkers, ECG, multimodality imaging, and endomyocardial biopsy; cTnI is more cardiac specific, though cTnT may carry greater prognostic value for major adverse cardiovascular events.
• Optimal on-treatment surveillance strategies remain an area of investigation.

## Acute Inpatient Management

### First-line therapy: high-dose corticosteroids

High-dose intravenous methylprednisolone is the cornerstone of acute management, with broad consensus across all major professional societies on early and intensive steroid therapy, though specific dosing and taper duration vary considerably (Table 2).^1^^,^^4^, ^5^, ^6^, ^7^, ^8^ Current dosing and tapering recommendations are based on expert consensus rather than randomized controlled trials. Prospective comparative studies are needed.

### Second-line agents

In steroid-refractory cases, defined by the IC-OS as worsening clinical deterioration and/or persistent troponin elevation despite high-dose methylprednisolone, second-line immunosuppressive agents are recommended.^6^ In addition, some experts advocate earlier introduction of second-line therapy in selected patients with features suggestive of worse outcomes (eg, reduced LVEF, wall motion abnormalities, markedly elevated troponin, significant conduction disease or ventricular arrhythmias, or triple M overlap syndrome, as reflected in the novel risk score developed by Power et al^27^), rather than awaiting refractory disease. This practice is reflected in IC-OS recommendations advising first-line combination therapy in severe/fulminant myocarditis or triple M overlap syndrome.^6^ Multiple agents have been used with selection guided by disease severity, overlap syndromes, and local expertise (Table 3).^6^ Supportive cardiac care should parallel immunosuppression, including pacing for advanced heart block, mechanical circulatory support for cardiogenic shock, and guideline-directed medical therapy for reduced LVEF.^6^ Individual agents are discussed in detail in “Outpatient Management of Steroid-Refractory ICI Myocarditis.”

**Table 3:** Immunosuppressive Therapies for Steroid-Refractory ICI Myocarditis

Agent	Mechanism of Action	Dosing	Key Monitoring	Level of Evidence			
				Case Series	Cohort/Meta-Analysis	RCT	Guideline
Abatacept	CTLA-4-Ig fusion protein; blocks CD80/CD86 co-stimulation, suppressing T-cell activation	10 mg/kg IV (ATRIUM protocol); up to 20 mg/kg	Infusion reactions, infection signs; CD86 receptor occupancy (investigational)	Salem et al^60^	Nielsen et al;^32^ Salem et al^15^	Ongoing: ATRIUM^57^	NCCN^7^: option if no response to steroids
Ruxolitinib	JAK1/JAK2 inhibitor; blocks JAK-STAT signaling, reduces inflammatory cytokines	5-10 mg orally twice a day (per case series; no guideline-specified dosage)	CBC (thrombocytopenia, anemia), LFTs	Nguyen et al^61^	Salem et al^15^	Ongoing: ACHLYS^58^	NCCN^7^: with abatacept for concomitant myositis/myocarditis
Mycophenolate mofetil	IMPDH inhibitor; blocks guanine nucleotide synthesis, suppresses B-/T-cell proliferation	0.5-1 g orally every 12 h (NCCN); titrate from 250 mg twice a day as tolerated	CBC (leukopenia), LFTs; GI side effects; delayed onset 2-4 wk	Wilson et al^62^	Nielsen et al^32^	—	NCCN^7^: option if no response to steroids
Tacrolimus	Binds FKBP, inhibits calcineurin, suppresses IL-2 and T-cell activation	0.05-0.1 mg/kg/d orally twice a day, titrate by trough drug levels	Drug trough levels, renal function, K^+^, Mg^2+^; extensive drug interactions	Sakaguchi et al^63^	Nielsen et al^32^	—	Second-line by ACC and AHA^5^
IVIG	Modulates B-/T-cell activation, Fc receptor blockade, autoantibody neutralization	2 g/kg IV divided over 2-5 d (NCCN)	Renal function (avoid sucrose-containing formulations), thrombotic risk	Sakaguchi et al^63^	Nielsen et al^32^	—	NCCN^7^: option if no response to steroids
Alemtuzumab	Anti-CD52; profound lymphocyte depletion via complement-dependent cytolysis and ADCC	Per institutional protocol (no standardized dosage)/IV	CBC (prolonged lymphopenia months–years), CMV/EBV viral loads, infection	Esfahani et al^64^	Nielsen et al^32^	—	NCCN^7^: option if no response to steroids; ACC^5^: hemodynamic instability
ATG	Polyclonal antithymocyte antibodies; T-cell depletion via complement lysis, ADCC, apoptosis	Per transplant/GCM protocols/IV	CBC (lymphopenia, thrombocytopenia), CMV/EBV viral loads; premedication for serum sickness	Tay et al^65^; Stein-Merlob et al^66^	Nielsen et al^32^	—	NCCN^7^: option if no response to steroids; AHA^49^: unstable steroid-refractory patients
Plasmapheresis	Removes circulating antibodies, immune complexes, cytokines; clears ICIs	1-1.5 plasma volumes per session (per standard practice)	Electrolytes, hemodynamics, coagulation; removal of therapeutic drugs requiring dose timing adjustment	Ke et al^46^; Stein-Merlob et al^66^	—	—	NCCN^7^: option if no response to steroids; IC-OS^6^:MG-like presentations
Tocilizumab	Anti-IL-6R; blocks JAK-STAT3 inflammatory cascade	8 mg/kg IV every 4 wk or 162 mg SC weekly/biweekly (FDA CRS/RA dosing)	LFTs, lipids, CBC (neutropenia), infection screening; GI perforation risk	Doms et al^19^	Fa'ak et al^51^	—	—
Tofacitinib	JAK1/JAK3 inhibitor; blocks JAK-STAT signaling	5 mg orally twice a day	CBC, LFTs, lipids; cardiovascular safety considerations	Wang et al^67^	Nagra et al^37^	—	—
Infliximab	Anti-TNF-α monoclonal antibody; neutralizes soluble and membrane-bound TNF-α	5 mg/kg IV at weeks 0, 2, 6, then every 8 wk	LFTs, CBC, infection; contraindicated in moderate to severe HF; monitor for infusion reactions	Padegimas et al^68^	Nielsen et al^32^	—	NCCN^7^: Option, use with caution in reduced LVEF

This table summarizes the mechanisms, dosing, monitoring requirements, and supporting evidence for immunosuppressive therapies used in steroid-refractory ICI-associated myocarditis. Several dosing regimens are derived from case series, transplant protocols, or investigational studies because standardized ICI myocarditis regimens remain unavailable. Supporting references are categorized by the publication type of the cited evidence (case report/series; cohort study or meta-analysis; randomized controlled trial; or professional society guideline recommendation). Case reports cited are not comprehensive. No completed randomized trial data exist for any agent; randomized trials of abatacept and ruxolitinib (ATRIUM, ACHLYS) are ongoing. Several agents are supported principally by the meta-analysis of Nielsen et al.,^32^ in which the pooled data are derived predominantly from individual case reports and small case series.

ACC = American College of Cardiology; ADCC = antibody-dependent cellular cytotoxicity; AHA = American Heart Association; ATG = antithymocyte globulin; CBC = complete blood count; CMV = cytomegalovirus; EBV = Epstein-Barr virus; FDA = Food and Drug Administration; GCM = giant cell myocarditis; GI = gastrointestinal; HF = heart failure; ICI = immune checkpoint inhibitor; IL = interleukin; IMPDH = inosine monophosphate dehydrogenase; IV = intravenous; IVIG = intravenous immunoglobulin; JAK = Janus kinase; LFT = liver function test; MG = myasthenia gravis; MMF = mycophenolate mofetil; NCCN = National Comprehensive Cancer Network; SC = subcutaneous; STAT = signal transducer and activator of transcription; TNF = tumor necrosis factor.

### Criteria for discharge and transition to outpatient care

No formal, validated discharge criteria exist specifically for ICI myocarditis. However, in our experience, transition to outpatient management should be considered when the following conditions are met: 1) hemodynamic stability without vasopressor or inotropic support; 2) declining or stable troponin trajectory on the current immunosuppressive regimen; 3) absence of high-grade arrhythmias or new conduction abnormalities for at least 48 to 72 hours; and 4) an established oral immunosuppressive regimen; and 5) a clearly defined outpatient follow-up plan, including scheduled biomarker surveillance, cardiology follow-up within 2 to 4 weeks, and patient education regarding concerning clinical symptoms and return precautions. The NCCN emphasizes appropriately timed follow-up visits with the oncologist and comanaging subspecialists, as these patients are likely receiving new immunosuppressive agents requiring further adjustment.^7^ Given that conduction abnormalities are associated with increased mortality in ICI myocarditis, ambulatory rhythm monitoring at discharge is recommended, in accordance with NCCN Guidelines and the 2024 American College of Cardiology (ACC) Expert Consensus Decision Pathway Document. For patients with implanted rhythm monitoring devices, more frequent interrogations (eg, monthly) post-discharge should be considered to monitor for ventricular and atrial arrhythmias. For patients without devices, extended ambulatory rhythm and event monitors can offer higher diagnostic yield than 24-hour rhythm monitoring. Ambulatory continuous cardiac/event monitors permitting real-time uploading of symptomatic or automatically triggered episodes should be considered for high-risk patients. In addition, patient education on warning symptoms and indications for urgent evaluation is an essential component of discharge planning.

**Summary**

• High-dose intravenous methylprednisolone is first-line therapy; steroid-refractory or fulminant cases require second-line immunosuppression.
• No validated discharge criteria exist; ambulatory rhythm monitoring at discharge is recommended for patients who develop new conduction abnormalities and/or atrial/ventricular arrhythmias with ICI myocarditis.

## Outpatient Management of Steroid-Refractory ICI Myocarditis

### Outpatient surveillance strategy

#### Risk stratification and prognostication

The IC-OS classification of smoldering, nonsevere, and severe (fulminant) myocarditis provides a clinically actionable framework for outpatient decision making.^6^ Patients initially classified as having nonsevere or severe myocarditis who have been stabilized and discharged should be reassessed at each outpatient visit to determine whether their disease has transitioned to a smoldering phenotype or has fully resolved. Conversely, a patient with smoldering myocarditis who develops new conduction abnormalities should be reclassified and managed accordingly. Patients can also be categorized using the Common Terminology Criteria for Adverse Effects grading system.

The International ICI-Myocarditis Registry risk score developed by Power et al^27^ provides further guidance in prognostication. Developed and validated as an inpatient triage tool using presenting clinical parameters, this score was derived from a multicenter registry of 748 patients across 17 countries. The 30-day incidence of the primary composite outcome (severe arrhythmia, heart failure, respiratory muscle failure, and/or cardiomyotoxicity-related death) was 33%, with cardiomyotoxicity-related death occurring in 13%. The risk score incorporates 5 parameters assessed at presentation: active thymoma (hazard ratio [HR]: 3.6), presence of cardiomuscular symptoms (HR: 2.6), low QRS voltage on presenting ECG (HR: 1.9 for ≤0.5 mV vs >1 mV), LVEF <50% (HR: 1.7), and incremental troponin elevation (HR: 1.8, 2.9, and 4.6 for 20-, 200-, and 2000-fold above the upper reference limit, respectively). The 30-day primary outcome incidence increased from 4% (risk score = 0) to 81% (risk score ≥4), and the score was externally validated in 2 independent French and U.S. cohorts.^27^

Although designed for initial risk stratification at presentation, this score has potential implications for outpatient management. In the prospective French validation cohort, all patients with a risk score of 0 were managed without immunosuppression and experienced no cardiomyotoxic events.^27^ The IC-OS position statement incorporates this finding, noting that patients with low-risk scores and smoldering myocarditis may be candidates for close monitoring without immunosuppression, potentially avoiding the infectious complications associated with prolonged steroid therapy.^6^ Further study, ideally in the form of randomized controlled trials with immunosuppressive/nonimmunosuppressive strategies, is warranted.

#### Biomarker, imaging, and integrated outpatient surveillance

Serial biomarker monitoring is essential for outpatient surveillance of ICI myocarditis. The 2025 ACC Concise Clinical Guidance highlights cardiac troponins as fundamental biomarkers in diagnosing ICI-associated myocarditis, with nearly all cases showing elevation.^5^ The NCCN suggests considering high-sensitivity troponin and N-terminal pro–B-type natriuretic peptide (NT-proBNP) at baseline and serially during treatment to detect abnormal biomarkers that may precede symptomatic myocarditis.^7^

Selecting between troponin T and troponin I is important for outpatient monitoring, although this may be dictated by local availability. cTnT normalizes more slowly (median 133 days) than cTnI (median 17 days).^24^ Persistent cTnT elevation may reflect ongoing skeletal muscle involvement rather than active myocardial inflammation, whereas cTnI is more specific for cardiac disease activity. This can be clinically helpful in determining disease course and/or severity; for instance, a rising cTnI during steroid taper likely indicates true myocardial recrudescence, whereas persistently elevated cTnT with concurrent myositis may not require increased cardiac immunosuppression.

For patients with established ICI myocarditis who have been discharged, the 2024 ACC Expert Consensus Decision Pathway on Strategies and Criteria for the Diagnosis and Management of Myocarditis provides the most relevant guidance.^28^ This document stratifies post-discharge surveillance by risk: patients with stage D or medium-/high-risk stage C myocarditis (defined as patients with any of the following: reduced LVEF, nonsustained ventricular tachycardia or higher-grade ventricular ectopy, significant bradyarrhythmias, late gadolinium enhancement on CMR, increased myocardial FDG uptake on PET, or hemodynamic instability/clinical heart failure) require the most intensive follow-up.

Close outpatient follow-up within 2 to 4 weeks post-discharge is recommended for all patients with established ICI myocarditis. For stage D or medium-/high-risk stage C patients, this visit should include biomarkers (high-sensitivity troponin, natriuretic peptides if elevated at baseline, and C-reactive protein if elevated at baseline), ECG, and echocardiography when feasible. For patients with symptomatic heart failure with reduced ejection fraction, an office visit should ideally occur within 1 week of hospital discharge. Interval visits focus on up-titration of guideline-directed medical therapy as appropriate. Post-discharge guidance on imaging surveillance by CMR or echocardiography is lacking, especially in patients with preserved LVEF. FDG-PET may have a role when CMR is equivocal or not feasible; the 2024 ACC Expert Consensus Decision Pathway notes it may provide additional sensitivity for identifying myocarditis of mild severity or prolonged duration, though no validated protocols exist to date to accurately identify active inflammation related to irAEs.^28^

During the early post-discharge period (weeks 1-2), the initial office visit serves as a critical checkpoint for detecting early deterioration and establishing baseline recovery parameters. The evaluation should include a comprehensive physical examination with attention to volume status and signs of heart failure decompensation; ECG; and biomarker assessment including high-sensitivity cardiac troponin (T and/or I), B-type natriuretic peptide or NT-proBNP, CPK (particularly if overlap myositis is suspected or confirmed), complete blood count (CBC), and comprehensive metabolic panel. Echocardiography should be performed in accordance with best practice standards in cardio-oncology (LVEF assessment by 3-dimensional echocardiography, biplanar quantification, speckle tracking if available).

During the intensive surveillance phase (weeks 2-6), weekly visits are recommended in our practice, and these may alternate between in-person and telehealth encounters depending on clinical stability. During this phase, we typically perform weekly high-sensitivity troponin (preferably troponin I) and CPK to guide immunosuppressive management, along with B-type natriuretic peptide or NT-proBNP, CBC, and comprehensive metabolic panel, continued weekly until clinical stability has been demonstrated. This frequency reflects the critical steroid taper period when biomarker rebound may signal inadequate immunosuppression. Clinicians should interpret troponin trends in the context of the differing normalization kinetics of cTnT and cTnI as described previously.

As biomarkers stabilize and steroid doses decrease during the stabilization phase (weeks 6-12), visits may be spaced further apart. The same laboratory panel should be obtained at each visit and can be discontinued once normal values and/or prolonged clinical stability has been achieved. Continued biomarker surveillance remains important to detect subclinical rebound while weaning immunosuppression. Repeat echocardiography should be considered approximately every 3 months, based on expert opinion, to assess for progressive deterioration of left ventricular function. Ambulatory rhythm monitoring should be considered if prior arrhythmias were documented; the role of wearable and implantable cardioverter-defibrillators is unclear, although these devices can be considered in patients who experienced significant ventricular arrhythmias in the acute phase of myocarditis, as the post-discharge risk of sudden cardiac death is unknown.

During long-term follow-up (months 3-6 and beyond), monthly visits facilitate the transition to routine cardio-oncology follow-up. Laboratory monitoring continues at each visit as clinically indicated. This phase focuses on optimizing guideline-directed medical therapy for heart failure if reduced ejection fraction persists, monitoring for late complications, and coordinating with an oncologist regarding alternative cancer therapies and potential ICI rechallenge assessment. The 6-month timepoint, albeit arbitrary, may serve as a reasonable juncture at which repeat echocardiography and/or multiparametric CMR with T1/T2 mapping and late gadolinium enhancement assessment can be considered for patients at medium to high risk, particularly given that the long-term risk of cardiomyopathy/heart failure in survivors of ICI myocarditis remains unknown.^28^ Emerging ICI-specific data are beginning to address this gap: in a prospective study of 62 patients undergoing serial CMR, baseline and follow-up parameters (LVEF, feature-tracking strain, left atrial strain) correlated with adverse cardiovascular events, suggesting that serial CMR may aid risk stratification, though larger studies are needed to guide management.^29^

Proposed clinical triggers for escalation of immunosuppression in the outpatient setting include the following: 1) a troponin increase greater than 50% from nadir during steroid taper; 2) new or worsening symptoms such as dyspnea, chest pain, palpitations, or syncope; 3) new ECG abnormalities, particularly conduction disturbances; and 4) inability to taper steroids without biomarker recrudescence. These thresholds are based on expert consensus rather than prospective validation, underscoring the need for further research (Figure 2, Central Illustration).^1^^,^^4^, ^5^, ^6^

**Figure 2:**
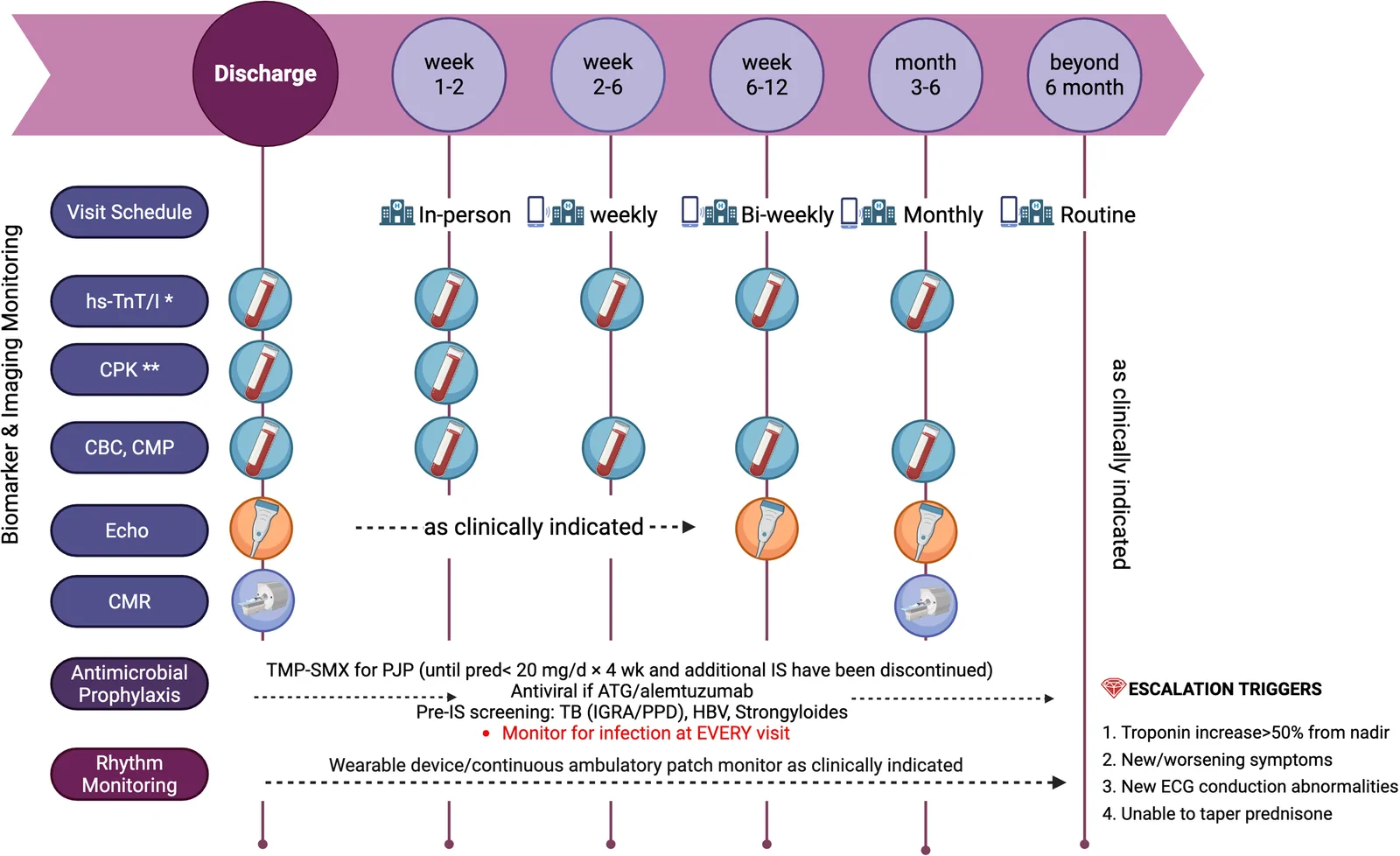
Proposed Integrated Outpatient Surveillance and Management Timeline for ICI-Associated Myocarditis Proposed post-discharge surveillance timeline for immune checkpoint inhibitor (ICI)–associated myocarditis based on our institutional experience. Escalation triggers include a >50% troponin increase from nadir, new or worsening symptoms, new electrocardiogram (ECG) conduction abnormalities, or inability to taper prednisone (pred). ∗Median normalization: 133 days for cardiac troponin T and 17 days for cardiac troponin I. ∗∗Monitor creatine phosphokinase (CPK) when myositis is suspected or confirmed. ATG = antithymocyte globulin; CBC = complete blood count; CMR = cardiac magnetic resonance; CMP = comprehensive metabolic panel; HBV = hepatitis B virus; hs-TnT/I = high-sensitivity cardiac troponin T/cardiac troponin I; IGRA = interferon gamma release assay; IS = immunosuppression; PJP = *Pneumocystis jirovecii* pneumonia; PPD = purified protein derivative; TB = tuberculosis; TMP-SMX = trimethoprim-sulfamethoxazole.

Created with biorender.com.

**Central Illustration:**
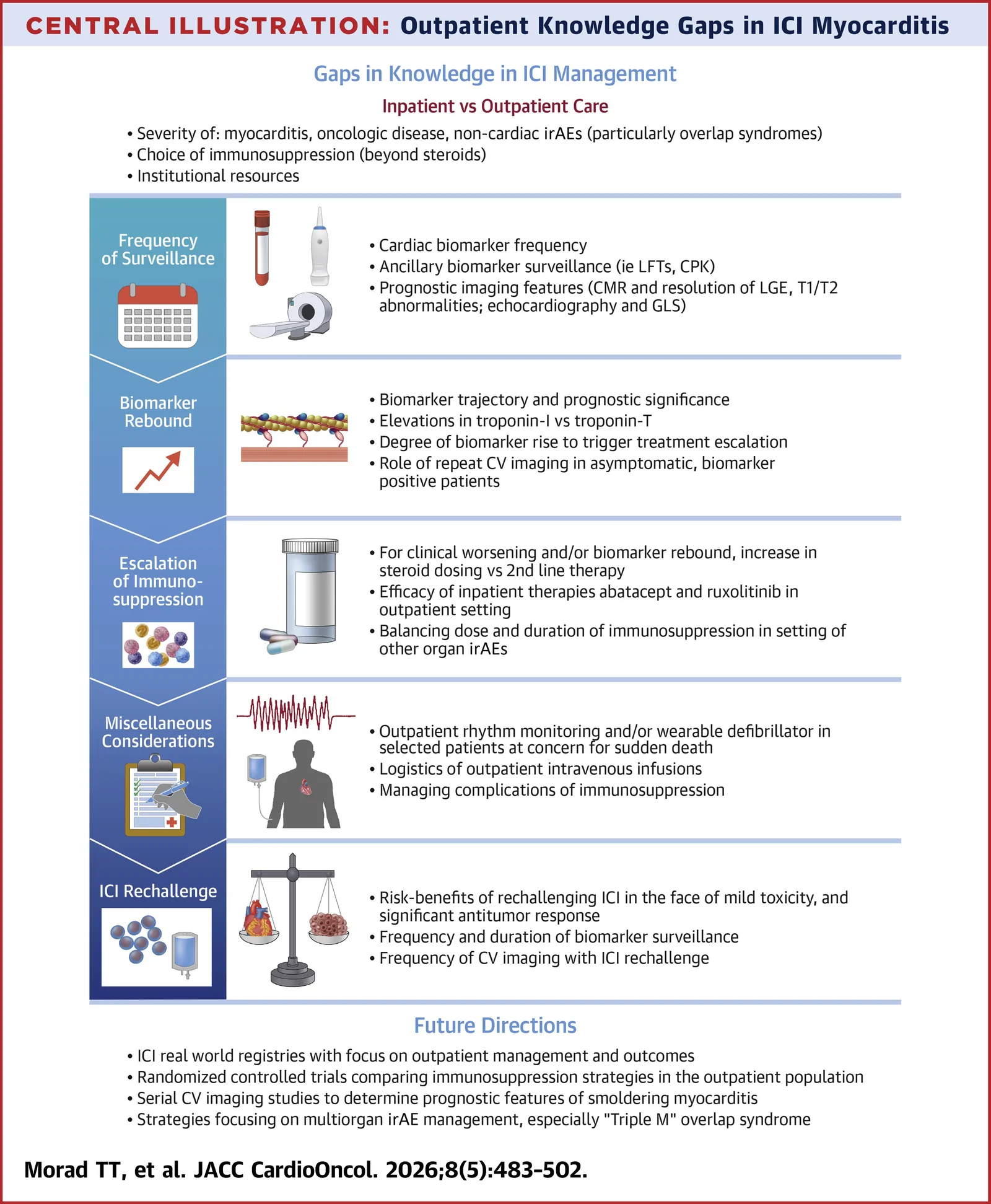
Outpatient Knowledge Gaps in ICI Myocarditis Uncertainties in outpatient immune checkpoint inhibitor (ICI) myocarditis care are summarized, including surveillance, biomarker rebound, immunosuppression escalation, logistical considerations, and ICI rechallenge. Factors influencing management are shown on the left, and research priorities are shown on the right. Arrows indicate transition from inpatient to outpatient care; the question mark denotes uncertainty regarding rechallenge. CMR = cardiac magnetic resonance; CPK = creatine phosphokinase; CV = cardiovascular; GLS = global longitudinal strain; irAE = immune-related adverse event; LFT = liver function test; LGE = late gadolinium enhancement; LVEF = left ventricular ejection fraction; T1/T2 = longitudinal and transverse relaxation parameters; Triple M = myocarditis, myositis, and myasthenia gravis–like illness.

Created with biorender.com.

#### Telehealth integration

Telehealth visits can be integrated into the post-discharge surveillance schedule for patients recovering from ICI-associated myocarditis. An American Heart Association (AHA) scientific statement supports telehealth for remote cardiovascular monitoring, including structured telephone/video support and wearable device integration.^30^ In this population, telehealth encounters are particularly well suited for symptom assessment, medication adherence review, discussion of laboratory results obtained at local facilities, and steroid taper guidance, thereby reducing patient burden while maintaining close surveillance.^30^

#### Patient education

Patients recovering from ICI-associated myocarditis should receive education tailored to their clinical needs. Key topics include: 1) recognizing symptoms such as chest pain, shortness of breath, palpitations, fainting, dysphagia, diplopia, ptosis, and weakness and understanding when to seek emergency care, as symptoms may be nonspecific and can recur; 2) adhering to prescribed medications, with emphasis on not adjusting steroid doses independently, as stopping steroids early can cause disease flare, and prolonged use may lead to complications such as glucocorticoid-induced adrenal insufficiency (distinguishing between ICI-associated adrenalitis and adrenal insufficiency also may require involvement of onco-endocrinology specialists); 3) identifying signs of infection, especially due to immunosuppression; 4) undergoing regular laboratory monitoring to guide immunosuppressive therapy adjustments; and 5) avoiding strenuous activity for 3 to 6 months, with return to activities guided by biomarker normalization, imaging, and exercise testing per general myocarditis guidelines. Patient and caregiver education on glucocorticoid risks is recommended,^31^ along with osteoporosis prophylaxis (vitamin D, calcium) and gastritis prophylaxis (proton pump inhibitor or H2 blockers) when indicated.

#### Coordination with oncology

Effective communication with the oncology team is essential and should focus on 3 areas: 1) ICI hold status and duration, as NCCN Guidelines recommend permanent discontinuation of immunotherapy for grades 2 to 4 myocarditis;^7^ 2) the possibility of ICI rechallenge in select cases depending on weighing risks and benefits of treatment efficacy against recurrent myocarditis risk;^7^ and 3) continuation of non-ICI–related cancer regimens and alternative cancer treatment options, which should be discussed in a multidisciplinary fashion between cardiovascular, cardio-oncology, and oncology care teams. ICI rechallenge may be considered on a case-by-case basis for low-severity myocarditis when no alternative cancer therapies are available.^5^ IC-OS consensus states that rechallenge should occur only when myocarditis has clinically resolved and immunosuppression is minimal (ideally <10 mg/day prednisone equivalent).^6^ Risk mitigation includes using monotherapy over combination regimens. Given the presumed heightened risk of recurrence, a strategy of enhanced cardiac surveillance should be implemented on rechallenge, including biomarker (troponin) and ECG assessment before each cycle of ICI therapy, although supporting evidence remains limited.

### Outpatient corticosteroid management

#### Oral prednisone taper strategies

Following initial stabilization with high-dose intravenous methylprednisolone, patients are transitioned to oral prednisone at 1 to 2 mg/kg/day once troponin concentrations trend downward. Steroid taper strategies vary across professional societies, as summarized in Table 2, with recommended taper durations ranging from approximately 4 to 6 weeks (American Society of Clinical Oncology, ESC) to 6 to 12 weeks (NCCN), alongside IC-OS advising a gradual reduction of roughly 10 mg/wk that is slowed to less than 10 mg/day and guided by troponin. Notably, these recommendations are based largely on expert consensus and extrapolation from the transplant rejection literature rather than prospective comparative data, and the optimal taper duration remains undefined. It is also important to frequently reassess and prioritize steroid treatment toward the dominant irAE process affecting the patient, which may pivot toward other disease processes, such as myositis, hepatitis, colitis, and other organ systems that may be affected.

#### Managing rebounding biomarkers/smoldering myocarditis

A particularly challenging scenario in outpatient management is the smoldering myocarditis phenotype. Decisions regarding escalation of immunosuppression, hospital readmission, or acceptance of biomarker abnormalities present a clinical dilemma that current guidelines do not adequately address.

A related but distinct challenge is the taper-flare phenomenon, in which patients demonstrate good initial clinical response to high-dose steroids with declining troponin concentrations, only to experience anywhere from mild to significant biomarker recrudescence while attempting to wean oral prednisone with no or unchanged symptoms. In contrast to smoldering myocarditis, which reflects persistent low-grade disease, the taper-flare phenomenon represents reactivation of inflammation unmasked by steroid withdrawal. Both scenarios, however, share the common feature of biomarker-symptom dissociation and require a similar management approach. Importantly, troponin elevation in this setting may have noninflammatory mimickers; patients with preexisting cardiovascular disease, chronic kidney disease, or heart failure or patients receiving concurrent cardiotoxic agents (eg, anthracyclines, trastuzumab, or tyrosine kinase inhibitors) may have chronically elevated troponin concentrations unrelated to active myocarditis, and establishing a new postevent baseline with management guided by relative changes rather than absolute values is essential in these populations.

When troponins increase during taper, a strategy is to increase the steroid dosage and prolong the tapering process. However, prolonged high-dose corticosteroid exposure increases the risk of opportunistic infections and metabolic complications and may delay resumption of oncologic therapy. In patients with recurrent biomarker elevation, clinical deterioration, or smoldering/taper-flare phenotypes, there is growing interest in steroid-sparing strategies using second-line agents such as ruxolitinib to shorten corticosteroid exposure in both inpatient and outpatient settings, although evidence comparing these strategies with steroid-intensive regimens is lacking.^15^

If troponin increases but the patient remains clinically stable, the following stepwise approach is recommended: 1) confirm with repeat testing; 2) obtain an ECG to assess for new conduction abnormalities; 3) consider echocardiography; 4) evaluate for intercurrent illness, renal dysfunction, or other noninflammatory causes; and 5) assess medication adherence. This evaluation distinguishes lower-risk patients (isolated, modest biomarker fluctuation, preserved LVEF, and no ECG or symptomatic changes), in whom continued close observation is appropriate, from higher-risk patients (rising biomarker kinetics, reduced or declining LVEF, new conduction disease or arrhythmias, or features of triple M overlap syndrome), in whom escalation of immunosuppression should be considered more readily. In our experience, small increases in troponin, which may occur with high-sensitivity assays, without clinical symptoms should not prompt the need to escalate steroid dosing and/or second-line treatments. For patients who experience significant biomarker recrudescence with other concerning clinical symptoms (ie, new cardiovascular symptoms, arrhythmias, heart failure), readministration of high-dose intravenous methylprednisolone (1 g/day for 3-5 days) may be considered. Hospital readmission should be considered for significant elevations (eg, greater than 50% increase from nadir, a threshold based on expert consensus rather than prospective validation), new symptoms, or new ECG abnormalities.

Our institutional practice does not escalate immunosuppression necessarily with mild fluctuations of biomarker levels of high-sensitivity troponin concentrations in the outpatient setting, and we continue tapering steroids if the patient is asymptomatic. If a clinically determined significant increase in biomarker levels occurs, further second-line immunomodulatory agents are considered (ie, ruxolitinib, mycophenolate, tacrolimus) with consultation with cardiomyopathy specialists. There is no defined cutoff of percentage of rise of cardiac biomarkers to change management, and surveillance and management strategies require further study.

#### Steroid-related complications during prolonged tapers

Prolonged corticosteroid therapy carries significant risks that must be actively monitored in the outpatient setting, including hyperglycemia, osteoporosis, adrenal insufficiency, and psychiatric effects. Steroid-induced myopathy warrants vigilance during prolonged high-dose therapy, as it can confound assessment of concurrent myositis; a rising or nonimproving CPK with new proximal weakness should prompt consideration of both active myositis and steroid myopathy, as management differs. For prophylaxis, the NCCN recommends vitamin D and calcium supplementation and notes that steroid use exceeding 30 mg daily for more than 30 days places patients at high risk for vertebral fractures.^7^ Proton pump inhibitor or H2 blocker therapy should be considered for patients at higher risk of gastritis for the duration of steroid therapy.

The management of steroid-refractory ICI myocarditis requires balancing adequate immunosuppression with the risk of opportunistic infections. The 2025 ACC Concise Clinical Guidance states that prophylaxis against opportunistic infections should be ensured during prolonged steroid tapers or when other immunosuppressive agents are used.^5^ As no ICI myocarditis–specific antimicrobial prophylaxis guidelines exist, treatment is modeled after the transplant and oncology literature. The NCCN recommends *Pneumocystis jirovecii* pneumonia prophylaxis with trimethoprim-sulfamethoxazole (preferred) for patients expected to receive ≥20 mg daily prednisone equivalent for ≥4 weeks, continuing until prednisone dosage is less than 20 mg/day for at least 4 weeks and additional immunosuppressants have been discontinued; for patients with sulfonamide allergy, atovaquone is a commonly used alternative in clinical practice, though the NCCN lists aerosolized or intravenous pentamidine as options, with caution regarding atovaquone (diarrhea risk) and dapsone (hemolytic anemia risk; glucose-6-phosphate dehydrogenase screening required).^7^ Pre-immunosuppression screening should include hepatitis B virus, HIV, hepatitis C virus, tuberculosis, and *Strongyloides* immunoglobulin G (IgG) serology for endemic regions.^6^ Herpes simplex virus/varicella-zoster virus prophylaxis should be considered with antithymocyte globulin (ATG) or alemtuzumab, and antifungal prophylaxis should be considered in endemic areas, with attention to azole–calcineurin inhibitor interactions. Clinical vigilance for infection should be maintained at every visit, with a low threshold for work-up in patients receiving steroids plus additional immunosuppressants.

### Second-line immunosuppressive agents

For patients with steroid-refractory or severe disease, second-line immunosuppressive agents are recommended. Many treatments are adapted from those used in organ transplant rejection, given overlapping mechanisms of impaired peripheral tolerance.^1^^,^^4^, ^5^, ^6^ Although no agent has randomized data to date and no head-to-head comparisons exist, the strength of supporting evidence varies substantially: abatacept and JAK inhibitors (ruxolitinib, tofacitinib) have the most robust support from larger case series and systematic reviews, with randomized abatacept trials ongoing, whereas the remaining agents rest on moderate retrospective cohorts or small series and case reports (Table 3). Agent selection should be guided by strength of evidence, disease severity, overlap syndromes, institutional availability/insurance coverage, and multidisciplinary input.

#### Abatacept

Abatacept is a CTLA-4 immunoglobulin fusion protein that downregulates T-cell activation by binding CD80/CD86 on antigen-presenting cells, thereby blocking the co-stimulatory signal required for T-cell activation.^15^^,^^32^ This mechanism directly counteracts the effects of anti-CTLA-4 checkpoint inhibitors and addresses the T cell–mediated pathophysiology of ICI myocarditis. Following intravenous administration, abatacept has a terminal half-life of approximately 13 to 16 days; however, a steady-state immunosuppressive effect is not achieved until after approximately 8 to 12 weeks of repeated dosing, and clinically meaningful CD86 receptor occupancy may take several days to weeks after the initial dose, which is a rationale for concurrent use of a faster-acting agent such as ruxolitinib to bridge this lag period.^33^

Abatacept has emerged as a preferred second-line agent based on nonrandomized data and case reports suggesting efficacy. In a systematic review by Nielsen et al,^32^ abatacept was administered to 12 patients with ICI myocarditis, with reported success in 11 patients (91.7%); only 1 patient died of myocarditis (8.3%). This success rate exceeds that of other second-line agents. Salem et al^15^ demonstrated that systematic screening for respiratory muscle involvement coupled with early abatacept (dose-adjusted using CD86 receptor occupancy) and ruxolitinib reduced myotoxicity-related fatality from 60% in a historical cohort to 3.4% in a subsequent cohort of 30 patients (*P* < 0.0001).

#### Ruxolitinib

Ruxolitinib modulates immune responses by inhibiting the JAK/signal transducers and activators of transcription (STAT) signaling pathway through binding to JAK1 and JAK2, thereby reducing the downstream production of inflammatory cytokines including the CXCR3-CXCL9/CXCL10 axis.^18^ Ruxolitinib is rapidly absorbed orally, achieving peak plasma concentration within 1 to 2 hours, with a short elimination half-life of approximately 3 hours; steady-state concentrations are reached by day 3 of twice-daily dosing, and maximal STAT3 phosphorylation inhibition occurs within 2 hours of administration.^34^ This rapid onset of action makes ruxolitinib well suited to bridge the delayed immunosuppressive effect of abatacept. In the Salem et al^15^ study, patients with severe ICI myocarditis received ruxolitinib for approximately 1 month in addition to abatacept, with a dramatic reduction in mortality. As an oral agent, ruxolitinib is well suited for outpatient use. Monitoring should include CBC (for cytopenias, particularly thrombocytopenia and anemia) and liver function tests. For patients treated beyond 2 weeks, gradual tapering is advisable, as abrupt discontinuation of JAK inhibitors may precipitate a cytokine rebound (withdrawal) syndrome. Ruxolitinib access may also be limited by cost and insurance coverage.

#### Tofacitinib

Tofacitinib and other JAK inhibitors represent an emerging class of agents for ICI myocarditis. Tofacitinib is a JAK1/JAK3 inhibitor (in contrast to ruxolitinib, which targets JAK1/JAK2) that blocks the JAK-STAT signaling pathway, reducing the production of inflammatory cytokines.^35^ Tofacitinib is rapidly absorbed orally with peak plasma concentrations within 0.5 to 1 hour and an elimination half-life of approximately 3 hours, necessitating twice-daily dosing; clinical immunosuppressive effects are observed within the first 1 to 2 weeks of therapy. A multicenter observational study by Liu et al^36^ evaluated 53 patients treated with tofacitinib for various irAEs (including myocarditis). Among 30 steroid-resistant patients, the clinical remission rate was 96.7%, and among 12 patients with steroid taper failure, remission was 100%, though these rates reflect the overall irAE cohort rather than myocarditis alone. A recent systematic review by Nagra et al^37^ identified 104 patients treated with JAK inhibitors for ICI-related irAEs, with tofacitinib accounting for 82% of cases and myocarditis being the most common indication (70%).

As an oral agent, tofacitinib is well suited for outpatient use. Monitoring should include CBC, liver function tests, and lipid panels. The cardiovascular safety profile of JAK inhibitors warrants consideration, as the ORAL Surveillance trial^38^ raised concerns about cardiovascular events with tofacitinib in patients with rheumatoid arthritis, though the relevance of these findings to short-term use in ICI myocarditis is uncertain. Similar concerns with ruxolitinib regarding access and cost are also an issue with tofacitinib.

#### Mycophenolate mofetil

MMF inhibits inosine 5′-monophosphate dehydrogenase, which prevents new synthesis of guanine nucleotides, thus reducing proliferation of B and T immune cells.^17^ This mechanism is adapted from its use in transplant rejection and autoimmune diseases. MMF is rapidly hydrolyzed to mycophenolic acid (peak 1-2 hours, half-life ∼17-18 hours), supporting twice-daily dosing. However, maximal lymphocyte suppression requires 1 to 2 weeks, with full therapeutic effect at 4 to 6 weeks; steroid taper should not be accelerated prematurely when initiating MMF.^39^ In the Nielsen et al^32^ systematic review, MMF was administered to 45 patients with ICI myocarditis, with a success rate of 64.4%. Case reports have described successful use at dosages of 1,000 mg twice daily for approximately 1 month.^40^ The NCCN recommends MMF at 0.5 to 1 g every 12 hours.^7^

Neutropenia is the most significant adverse effect, occurring most frequently between days 31 and 180. Per FDA labeling, CBC should be obtained weekly in month 1, twice monthly in months 2 and 3, and monthly thereafter; dosage adjustments may be necessary for cytopenias.^41^ Gastrointestinal side effects are common; conversion to enteric-coated mycophenolate sodium (Myfortic) may improve tolerability in affected patients.^42^ MMF-associated colitis, though rare, should be considered in the differential diagnosis of new-onset diarrhea, given overlap with ICI-associated colitis.

#### Tacrolimus

Tacrolimus binds to FK506 binding proteins, which inhibits calcineurin and suppresses the transcription of proinflammatory cytokines such as IL-2, ultimately reducing T-cell activation and clonal expansion.^16^ This mechanism is adapted from its use in cardiac allograft rejection. Oral tacrolimus reaches peak concentrations within 1 to 3 hours with a half-life of approximately 12 hours (range: 4-41 hours); this is highly variable due to CYP3A4/CYP3A5 metabolism. The narrow therapeutic index of tacrolimus necessitates close trough-level monitoring, with attention to drug interactions, dietary factors, and hepatic or renal impairment.^43^ In the Nielsen et al^32^ systematic review, tacrolimus was administered to 6 patients with ICI myocarditis; 4 (66.7%) patients showed improvement, and 1 (16.7%) patient died. The 2025 ACC Concise Clinical Guidance lists tacrolimus among the additional immunosuppressive agents that can be considered.^5^ Similarly, the NCCN Guidelines for ICI-related hepatotoxicity reference tacrolimus use with monitoring of trough levels; however, the guidelines notably state there is no target tacrolimus trough level and recommend targeting the lowest dosage that induces a biochemical response.^7^ Side effects include nephrotoxicity, neurotoxicity (headache, tremors), electrolyte disturbances, and leukopenia/anemia.

#### Intravenous immunoglobulin

Intravenous immunoglobulin (IVIG) is indicated for myasthenia gravis–like presentations that can accompany myocarditis in triple M overlap syndrome.^22^ IVIG modulates the immune system through multiple mechanisms, including modification of B- and T-cell activation and differentiation, Fc receptor blockade, and neutralization of autoantibodies.^44^ IVIG achieves peak serum IgG levels immediately after infusion completion, with immunomodulatory effects typically observed within 24 to 72 hours; the elimination half-life of infused IgG is approximately 21 to 35 days, which supports its prolonged duration of effect.^44^ The NCCN recommends IVIG at 2 g/kg divided into equal doses given over 2 to 5 consecutive days.^7^ In the Nielsen et al^32^ systematic review, IVIG was administered to 63 patients with myocarditis, and improvement in clinical condition, cardiac biomarkers, and LVEF was noted in 58.7% of patients.

For outpatient administration, IVIG requires coordination with an infusion center. Considerations include renal toxicity (particularly in patients with preexisting renal impairment or volume depletion), thrombotic risk, and significant cost. Premedication with acetaminophen, methylprednisolone, and diphenhydramine is recommended to reduce infusion reactions. Of note, IVIG should be temporally separated from other antibody-mediated therapies to prevent accelerated clearance of antibody therapies.

#### Plasmapheresis/therapeutic plasma exchange

Plasmapheresis/therapeutic plasma exchange removes circulating antibodies, immune complexes, and cytokines from the blood and may also promote rapid clearance of circulating ICIs. The 2025 ACC Concise Clinical Guidance notes that in addition to removing pathologic antibodies, plasmapheresis may promote rapid clearance of circulating drugs, including immunosuppressive agents that may impede their effects.^5^ This is particularly relevant given the long half-lives of ICI antibodies (nivolumab ∼25 days, pembrolizumab ∼22 days, ipilimumab ∼15 days).^45^ The IC-OS position statement recommends IVIG and/or plasma exchange especially for myasthenia gravis–like presentations in the triple M overlap syndrome.^6^ A case series by Ke et al^46^ reported favorable outcomes in 4 patients treated with plasma exchange plus glucocorticoids for anti-PD-1/anti-PD-L1–induced myocarditis, with all patients improving and being discharged.

Plasmapheresis requires vascular access and specialized equipment, making it primarily an inpatient or specialized outpatient procedure. For outpatient continuation, it can be performed at apheresis centers, though logistical coordination is required. Important considerations include the need for central venous access, hypotension during procedures, electrolyte disturbances, increased bleeding risk, increased infection risk, and the removal of therapeutic drugs (including immunosuppressive agents), which may necessitate dosage timing adjustments.

#### Alemtuzumab

Alemtuzumab targets CD52, a glycoprotein expressed on the surface of mature lymphocytes, monocytes, macrophages, and natural killer cells, resulting in profound and prolonged lymphocyte depletion through complement-dependent cytolysis and antibody-dependent cellular cytotoxicity.^47^^,^^48^ Following intravenous administration, alemtuzumab produces rapid lymphocyte depletion within days, with near-complete T- and B-cell depletion typically occurring by the end of a treatment course; the elimination half-life is approximately 2 weeks, but lymphocyte recovery is markedly prolonged, with CD4^+^ T-cell counts often remaining suppressed for 12 months or longer.^47^^,^^48^ Evidence for alemtuzumab in ICI myocarditis is limited to case reports and expert opinion, with only 1 patient reported in the Nielsen et al^32^ systematic review; nonetheless, it is listed among agents that can be considered for refractory cases with hemodynamic instability by both the 2025 ACC Concise Clinical Guidance^5^ and the AHA scientific statement on fulminant myocarditis.^49^ Given its profound immunosuppressive effects, including prolonged lymphopenia lasting months to years, alemtuzumab is typically reserved for the most refractory and life-threatening cases. It is administered intravenously and requires close monitoring for infusion reactions, cytopenias, and opportunistic infections.

#### Antithymocyte globulin

ATG is a polyclonal antibody preparation derived from immunization of rabbits or horses with human thymocytes, resulting in antibodies that target multiple T-cell surface antigens and cause T-cell depletion through complement-dependent lysis, antibody-dependent cell-mediated cytotoxicity, and apoptosis.^50^ Rabbit ATG (Thymoglobulin) achieves significant T-cell depletion within 24 hours of the first dose; the serum half-life is approximately 2 to 3 days, though functional lymphocyte suppression persists for weeks to months.^50^ Its use in ICI myocarditis is adapted from its established role in treating cardiac allograft rejection and giant cell myocarditis, where it is used in combination with calcineurin inhibition (cyclosporine, tacrolimus) and corticosteroids to block T-cell infiltration of the myocardium.^48^ The AHA scientific statement recommends that ATG or IVIG can be considered in patients who are unstable and do not respond to corticosteroids.^49^ The IC-OS position statement recommends ATG as one of the first-line combination options with high-dose steroids for severe or fulminant myocarditis and/or triple M overlap syndrome.^6^

The indication and efficacy of use of ATG in the outpatient setting is unclear. The high mortality rate likely reflects its use in the most severe and refractory cases rather than inherent inefficacy. ATG requires intravenous administration, typically over several days, with premedication to prevent serum sickness and anaphylaxis. Outpatient monitoring should include CBC (for profound lymphopenia and thrombocytopenia), cytomegalovirus and Epstein-Barr virus viral load monitoring, and vigilance for opportunistic infections.

#### Tocilizumab

Tocilizumab has emerged as a promising agent for ICI myocarditis based on recent data. Tocilizumab blocks IL-6 signaling by binding to both soluble and membrane-bound IL-6 receptors, thereby inhibiting the JAK-STAT3 inflammatory cascade.^19^ Following intravenous administration, tocilizumab produces rapid suppression of C-reactive protein and IL-6-mediated signaling within 24 hours; the elimination half-life is concentration dependent, ranging from approximately 11 to 13 days at therapeutic doses.^19^ IL-6 is significantly elevated in patients with ICI myocarditis compared with baseline in patients with cancer before ICI initiation.^19^ A multicenter retrospective study by Fa'ak et al^51^ evaluated 92 patients treated with anti-IL-6R antibodies for various irAEs, reporting that 73% showed resolution or improvement to grade 1 or less, with no reduction in overall tumor response rate.

Tocilizumab is approved by the FDA for chimeric antigen receptor T-cell–induced cytokine release syndrome, providing a precedent for its use in immune-mediated cardiac inflammation.^52^ For outpatient use, tocilizumab can be administered intravenously at infusion centers or subcutaneously. Monitoring should include liver function tests, lipid panels, CBC (for neutropenia), and screening for infections. The risk of gastrointestinal perforation should be assessed, particularly in patients with a history of diverticular disease. A QuantiFERON-TB Gold (Qiagen) test for tuberculosis infection should be done before initiation of therapy.

#### Infliximab

Infliximab is a chimeric monoclonal antibody that binds soluble and transmembrane tumor necrosis factor alpha, a cytokine implicated in the pathogenesis of ICI myocarditis. Infliximab achieves peak serum concentrations immediately following intravenous infusion with an elimination half-life of approximately 7 to 12 days. However, its use in ICI myocarditis remains controversial. Infliximab has been associated with worsening heart failure in patients with preexisting moderate to severe heart failure (NYHA functional class III/IV, LVEF ≤35%) in the ATTACH trial,^53^ and the 2025 ACC Concise Clinical Guidance and NCCN advise caution owing to this risk.^5^^,^^7^ In the Nielsen et al^32^ systematic review, infliximab was administered to 21 patients, with 8 (38.1%) showing improvement and 8 (38.1%) dying of a cardiac event. Given these data, the role of infliximab in ICI myocarditis remains uncertain, and its use should be weighed carefully against the potential for cardiac decompensation, particularly in patients with reduced ejection fraction.

**Summary**

• A structured surveillance timeline with serial biomarkers, imaging, and defined escalation triggers is proposed.
• Second-line agents vary in onset of action, route, and monitoring requirements, guiding outpatient selection.
• Antimicrobial prophylaxis modeled after transplant protocols is essential during prolonged immunosuppression.

## Emerging therapeutic targets and ongoing trials

### Novel mechanistic targets

Several preclinical discoveries have identified promising therapeutic targets that may transform the management of ICI myocarditis. In preclinical models, selective TNFR2 blockade prevented cardiotoxicity while preserving antitumor efficacy, offering a potential strategy to uncouple cardiac injury from the therapeutic benefits of immunotherapy.^54^ An antibody targeting CXCR6 prevented premature lethality, attenuated arrhythmias, and reduced histologic severity of myocarditis in *Lag3*^*−/−*^*Pdcd1*^*−/−*^ mice.^55^ The CXCR3–CXCL9/CXCL10 axis, central to the T cell–macrophage inflammatory loop, is another emerging target; antibody blockade of CXCR3 attenuated myocarditis in preclinical models, and interferon gamma blockade similarly reduced the expansion of CXCL9/CXCL10 macrophages.^14^ Pharmacologic gasdermin E (GSDME) blockade attenuated ICI myocarditis and improved long-term survival in mice by interrupting the pyroptosis cyclic GMP-AMP synthase–stimulator of interferon genes axis.^56^ Each of these targets addresses a specific component of the pathogenic cascade and could potentially be developed as outpatient-administered therapies.

### Ongoing clinical trials

Ongoing randomized clinical trials are evaluating the efficacy of abatacept and/or ruxolitinib in reducing major cardiovascular events in patients with ICI myocarditis in both inpatient and outpatient settings. The ATRIUM trial (NCT05335928)^57^ is a randomized, double-blind, placebo-controlled trial evaluating abatacept vs placebo in hospitalized patients with ICI-associated myocarditis. The ACHLYS trial (NCT05195645) is a phase 2, randomized, double-blind, dose-finding trial evaluating 3 abatacept doses (10, 20, or 25 mg/kg) combined with ruxolitinib and corticosteroids for severe or corticosteroid-resistant ICI myocarditis.^58^ Results from these trials will provide the first randomized evidence to guide second-line therapy. Whereas neither the ATRIUM nor the ACHLYS trial specifically addresses outpatient strategies, their follow-up data will inform prognostication and surveillance intensity. The absence of trials evaluating outpatient-specific management strategies remains a critical gap.

**Summary**

• Preclinical targets including *TNFR2*, *CXCR6*, and *GSDME* blockade may yield future outpatient therapies.
• The ATRIUM and ACHLYS trials will provide the first randomized evidence for second-line agents.

## Knowledge Gaps and Future Directions

Despite the growing body of literature on ICI myocarditis, significant knowledge gaps remain, particularly regarding outpatient management. Prospective registries specifically tracking outpatient ICI myocarditis management are needed to establish evidence-based protocols. Standardized definitions of steroid-refractory disease, including specific biomarker thresholds for escalation, require prospective validation. Head-to-head comparisons of second-line agents are lacking; the ATRIUM^57^ and ACHLYS^58^ trials will provide important data on abatacept, but comparative effectiveness studies of other agents are needed. Optimal antimicrobial prophylaxis protocols for patients with ICI myocarditis receiving multiagent immunosuppression have not been established. The role of wearable devices and remote monitoring technologies for continuous arrhythmia surveillance in this population warrants investigation. Patient-reported outcomes and quality-of-life data are conspicuously absent from the current literature. Finally, the application of artificial intelligence for biomarker trend analysis and risk prediction may enhance outpatient decision making but requires development and validation.

**Summary**

• Prospective registries, head-to-head agent comparisons, and outpatient-specific trial designs are urgently needed.
• Standardized biomarker thresholds for immunosuppressive escalation require prospective validation.

## Conclusions

ICI myocarditis remains one of the most challenging complications in contemporary cardio-oncology. Though acute inpatient management has become increasingly well defined, outpatient management of steroid-refractory disease represents a critical gap in current practice. This review provides a comprehensive synthesis spanning pathophysiology, diagnosis, acute management, and, most critically, outpatient management of steroid-refractory disease. The proposed framework, incorporating biomarker-guided steroid tapering, stepwise immunosuppressive escalation, structured surveillance, antimicrobial prophylaxis, and dynamic risk assessment, aims to provide clinicians with a practical roadmap for navigating this complex clinical scenario. As the number of patients receiving ICIs increases, the need for evidence-based outpatient management protocols will also increase. Collaborative, multicenter efforts to prospectively validate the strategies proposed in this review are needed.

## Funding Support and Author Disclosures

Dr Yang has received research grant funding from Amgen, Bristol Myers Squibb, Janssen Research and Development, and Novo Nordisk; consulting fees from Astellas and Edwards Lifesciences; advisory board fees from Skribe Medical; and speaker honoraria from Zoll Medical. Dr Srivastava has received speaker honoraria from Bayer and BridgeBio. All other authors have reported that they have no relationships relevant to the contents of this paper to disclose.
